# Vitamin A deficiency in critically ill children with sepsis

**DOI:** 10.1186/s13054-019-2548-9

**Published:** 2019-08-01

**Authors:** Xuepeng Zhang, Kaiying Yang, Linwen Chen, Xuelian Liao, Liping Deng, Siyuan Chen, Yi Ji

**Affiliations:** 1 Pediatric Intensive Care Unit, Department of Critical Care Medicine, West China Hospital of Sichuan University, Chengdu, 610041 China; 2 Division of Oncology, Department of Pediatric Surgery, West China Hospital of Sichuan University, Chengdu, 610041 China; 3 College of Clinical Medicine, North Sichuan Medical College, Nanchong, 637000 China; 4 Department of Pharmacy, Yiling Hospital, Yichang, 443100 China

**Keywords:** Sepsis, Pediatrics, Vitamin A, Vitamin A deficiency

## Abstract

**Background:**

Data that indicate vitamin A status in critically ill children with sepsis are sparse. The association between serum vitamin A levels and the clinical outcomes of sepsis has not been well assessed. The aim of this study was to assess the prevalence of vitamin A deficiency in critically ill children with sepsis and its association with clinical outcomes.

**Methods:**

Critically ill children with sepsis admitted to the pediatric intensive care unit were engaged in this prospective study. Sex- and age-matched approximate-health children from the Department of Pediatric Surgery were enrolled as the control group. Blood samples were collected from all patients in the first 24 h of admission for the measurement of serum vitamin A status. We compared vitamin A status between the sepsis group and the control group. In addition, we compared the clinical characteristics of the two subgroups of septic patients with vitamin A deficiency and those without vitamin A deficiency. Univariate and multivariable methods were used to evaluate the association between vitamin A deficiency and septic shock.

**Results:**

One hundred sixty septic children and 49 approximate-health children were enrolled in this study. Vitamin A deficiency was found in 94 (58.8%) subjects in the study group and 6 (12.2%) subjects in the control group (*P* < 0.001). In septic patients, 28-day mortality and hospital mortality in patients with vitamin A deficiency were not significantly higher than that in patients without vitamin A deficiency (*P* > 0.05). However, vitamin A levels were inversely associated with higher PRISM scores in septic children with VAD (*r* = − 0.260, *P* = 0.012). Vitamin A deficiency was associated with septic shock with an unadjusted odds ratio (OR) of 3.297 (95% confidence interval (CI), 1.169 to 9.300; *P* = 0.024). In a logistic model, vitamin A deficiency (OR, 4.630; 95% CI, 1.027–20.866; *P* = 0.046), procalcitonin (OR, 1.029; 95% CI, 1.009–1.048; *P* = 0.003), and the Pediatric Risk of Mortality scores (OR, 1.132; 95% CI, 1.009–1.228; *P* = 0.003) were independently associated with septic shock.

**Conclusion:**

The prevalence of vitamin A deficiency was high in children with sepsis. Vitamin A deficiency may be a marker of mortality in critically ill children with sepsis.

**Trial registration:**

Clinicaltrials.gov, NCT03598127

**Electronic supplementary material:**

The online version of this article (10.1186/s13054-019-2548-9) contains supplementary material, which is available to authorized users.

## Background

Vitamin A (VA) and its derivatives, all of which are a group of unsaturated nutritional organic compounds, play essential roles in embryonic development, growth, vision, reproduction, and the immune system [1–3]. Retinoic acid (RA), an active metabolite of VA, has been reported to promote anti-inflammatory regulatory T cell (Treg) differentiation and inhibit interleukin (IL)-6-induced proinflammatory T helper 17 (Th17) cells, which could balance pro- and anti-inflammatory immunity [4]. In addition to nyctalopia, which is the well-known manifestation of profound VA deficiency (VAD), extensive literature has provided evidence that VAD is associated with adverse health outcomes due to an increased risk of infection in children. VAD could impact immunity at multiple levels, including disturbing the integrity of the gastrointestinal mucosal barrier, decreasing monocyte and natural killer (NK) cell numbers, and impairing the function of macrophages, dendritic cells, and neutrophils [1, 5–7]. Our previous study revealed that VAD was associated with decreased concentrations of interferon-α (IFN-α) and enterovirus 71 (EV71) immunoglobulin M (IgM), resulting in decreased immunity and increased illness severity in children with EV71 infection [8, 9]. Although VAD has been an issue of concern for decades in the general populations, there are no available data regarding VA status in critically ill children with sepsis.

Sepsis, a life-threatening organ dysfunction caused by a dysregulated host response to infection, contributes to millions of deaths worldwide each year, with a mortality rate of more than 25%. Remarkably, sepsis is a common cause of death in children. The mortality of severe sepsis was reported to be as high as 34.6% in children [10]. It has been revealed that over 50% of deaths in preschool children were due to severe infectious diseases that could result in sepsis [11]. A steady increase in the incidence of severe sepsis has been reported in the past decades [12]. As a public health problem, sepsis has posed a significant burden on extensive health care resources for many years. It is reported as a complicated immune disorder characterized by both a hyperinflammatory immune response in the early stage and immunosuppression in the later stage [13–15]. Most deaths from sepsis occur due to opportunistic pathogen superinfections or latent viral reactivation resulting from immunosuppression [16].

VA is an immunomodulatory, and its deficiency may cause an imbalance between pro- and anti-inflammatory factors and impaired immune function, which are found in sepsis. There is a biological rationale that VAD may be a contributing factor related to poor clinical outcomes in patients with sepsis. Importantly, VAD is highly prevalent in children, especially in preschool children. However, there is a paucity of data regarding the correlation between VAD and sepsis. We hypothesize that VAD may play an important role in the pathogenesis and progression of sepsis in children. Therefore, the aim of the present study was to assess the prevalence of VAD in critically ill children with sepsis and the association between VAD and clinical outcomes.

## Method

This prospective study was conducted at the West China Hospital of Sichuan University from February 2018 to January 2019. The study was approved by the Ethics Committee of the West China Hospital of Sichuan University and was conducted in compliance with the Declaration of Helsinki. Prior to the initiation of study-related procedures, the legal guardians of the children were informed about the study and they provided the written informed consent. Patients from 0 months to 192 months who were admitted to the pediatric intensive care unit (PICU) with sepsis (as defined by *International pediatric sepsis consensus conference: Definitions for sepsis and organ dysfunction in pediatrics*) were consecutively enrolled [17]. Sex- and age-matched approximate-health children without sepsis were recruited from the Department of Pediatric Surgery as a control group. Approximately healthy children were defined as patients who received routine pediatric surgery, including circumcision, inguinal hernia repair, or tumorectomy of small-sized benign tumors. Criteria for exclusion were premature infants and low birth weight (LBW) infants, age > 18 years, condition of underlying organ dysfunction, having received chemotherapy or radiotherapy, hematological malignancies, primary or acquired immunodeficiency, and discharge against medical advice with an uncertain prognosis. No additional interventions were performed on those children. The legally authorized representatives of the children were not aware of their VA levels. All therapists were blinded to the study. This trial was registered on the public database ClinicalTrials.gov (NCT03598127).

Blood samples were collected from all patients during the first 24 h of admission before enteral nutrition and/or parenteral nutrition. VA is light-sensitive. Therefore, venous blood samples were immediately delivered into aluminum foil-wrapped tubes after collection. Next, the samples were centrifuged at 3000 rpm for 5 min to separate the serum. The serum was aliquoted in marked Eppendorf test tubes and frozen at − 80 °C until VA concentrations of the serum samples were analyzed by high-performance liquid chromatography. Serum VA levels below 20 μg/dl were considered deficient [18]. Demographic data of all recruited patients were recorded upon admission. The following data were collected from patients with sepsis: Pediatric Risk of Mortality (PRISM) scores (within the first 24 h of admission), temperatures, lactate levels, and basic hematological and biochemical test results. Clinically relevant data in sepsis patients, including the source of infection, positive blood culture, duration of mechanical ventilation, length of PICU stay, onset of severe sepsis or septic shock, mortality on discharge, and 28-day mortality, were collected. According to the International Consensus definition [17], sepsis with the development of acute respiratory distress syndrome, cardiovascular organ dysfunction, or two or more other acute organ dysfunctions was defined as severe sepsis. Sepsis with cardiovascular dysfunction was defined as septic shock. Organ dysfunction was also defined according to the International Consensus [17].

Statistical analyses were conducted using SPSS 22.0 for Windows (SPSS Inc., Chicago, IL, USA). Data with a normal or nonnormal distribution were described as the mean and standard deviation (SD) or median with 25% and 75% quartiles (interquartile range), respectively. Categorical variables were expressed as counts (percentages). Continuous variables with normal distributions were analyzed by using Student’s *t* test. *P* values were adjusted by the Bonferroni correction method when performing multiple *t* tests. Data without normal distributions were analyzed by a nonparametric test (Mann-Whitney *U* test). The chi-squared test or the Fisher exact test was used to analyze categorical data. Correlation between variables was tested by Pearson correlation. We used univariate and multivariable methods to evaluate the association between vitamin A deficiency and septic shock. A *P* value < 0.05 was considered statistically significant. Factors with *P* < 0.10 in the univariate analysis were analyzed in a multivariable regression analysis.

## Results

**Fig. 1:**
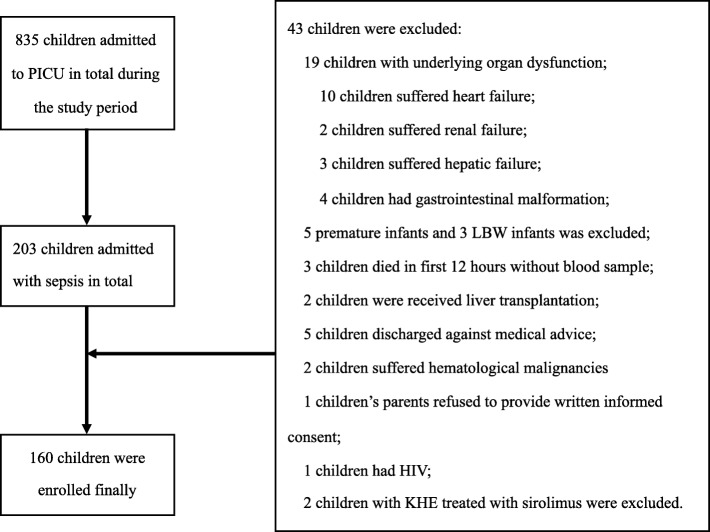
Screening and enrollment (HIV, human immunodeficiency virus; KHE, kaposiform hemangioendothelioma)

A total of 203 children were admitted with sepsis during the study period, and 160 patients with sepsis were finally enrolled in this study (Fig. 1). Forty-nine approximate-health control subjects from the Department of Pediatric Surgery were also included. The demographic characteristics of the sepsis group and control group are listed in Table 1. There was no significant difference in the demographic characteristics between the two groups. The mean VA status in septic children was significantly lower than that in the control group (0.192 ± 0.106 mg/L vs. 0.339 ± 0.119 mg/L; *P* < 0.001). The prevalence of VAD was 58.8% in the sepsis group and 12.2% in the control group, *P* < 0.001. In the sepsis group, 56.9% were boys, the median age was 12 months, and 77.5% of the children were younger than 60 months. In septic patients with VAD, 76.6% were younger than 60 months. The most likely source of infection was the gastrointestinal system, followed by the pulmonary system. In total, 29 patients (18.1%) suffered from severe sepsis, and 15.6% of patients had septic shock. In the subgroup of severe sepsis and septic shock, the rates of VAD were 79.3% and 80.0%, respectively (Additional file 1 and Table 3).

**Table 1:** Baseline characteristics

	Sepsis group	Control group	*P* values
	*N* = 160	*N* = 49	
Age, months	12.00 (3.00, 49.75)	11.00 (4.00, 62.00)	0.782^a^
Age, *n* (%)			0.460^b^
< 1 year	77 (48.1%)	26 (53.1%)	
1 year≤, < 5 years	47 (29.4%)	10 (20.4%)	
5 years≤	36 (22.5%)	13 (26.5%)	
Gender			0.294^b^
Male, *n* (%)	91 (56.9%)	32 (65.3%)	
Race, *n* (%)			0.299^c^
Tibetan	15 (9.4%)	3 (6.1%)	
Han	132 (82.5%)	45 (91.8%)	
Other	13 (8.1%)	1 (2.0%)	
Weight, kg	7.75 (4.00, 14.86)	9.00 (5.75, 16.75)	0.216^a^
Setting, *n* (%)			0.652^b^
Rural	79 (49.4%)	26 (53.1%)	
City	81 (50.6%)	23 (46.9%)	
Mean VA status, mg/L			
Total	0.192 (0.106)	0.339 (0.119)	0.000^d#^
< 1 year	0.192 (0.1043)	0.305 (0.131)	0.000^d^
1 year≤, < 5 years	0.189 (0.095)	0.401 (0.093)	0.000^d^
5 years≤	0.198 (0.125)	0.361 (0.091)	0.000^d^
VAD, *n* (%)	94 (58.8%)	6 (12.2%)	0.000^b^
< 1 year	45 (47.9%)	6 (100.0%)	
1 year≤, < 5 years	27 (28.7%)	0	
5 years≤	22 (23.4%)	0	
Source of infection, *n* (%)			
Respiratory system	40 (25.0%)		
Gastrointestinal system	73 (45.6%)		
Central nervous system	1 (0.6%)		
Blood stream	8 (5.0%)		
Soft tissue	10 (6.3%)		
Other	20 (12.5%)		
Respiratory and gastrointestinal system	5 (3.1%)		
Respiratory and central nervous system	2 (1.3%)		
Severe sepsis, *n* (%)	29 (18.1%)		
Septic shock, *n* (%)	25 (15.6%)		
Length of stay, days			
PICU	6.70 (3.50, 12.87)		
Hospital	14.65 (8.93, 22.80)		
PRISM	11.00 (7.00, 17.00)		
Mortality, *n* (%)			
Hospital	7 (4.4%)		
28-day	11 (6.9%)		
Time of ventilation, hours	24.00 (7.25, 117.50)		
Positive blood culture, *n* (%)	14 (8.75%)		

Data are presented as means (SD), median (quartile 1, quartile 3) or number (percentage)

*VA* vitamin A, *VAD* vitamin A deficiency, *PICU* pediatric intensive care unit, *PRISM* Pediatric Risk of Mortality score

^a^Mann-Whitney *U* test

^b^Chi-squared test

^c^Fisher exact test

^d^Student’s *t* test

^#^*P* < 0.0001

**Fig. 2:**
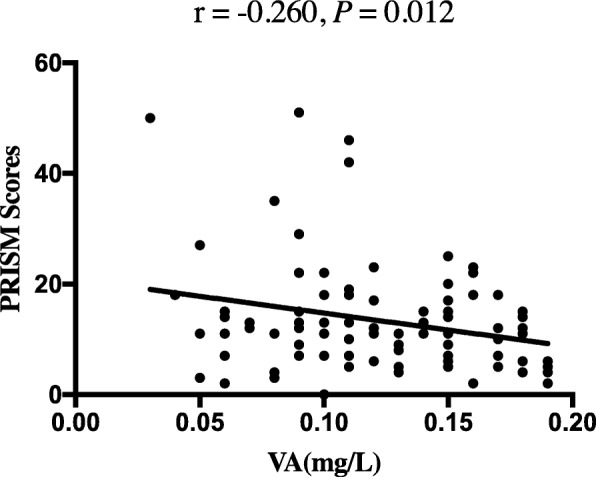
The correlation between serum vitamin A levels and PRISM scores in septic children with vitamin A deficiency. The concentrations of vitamin A were negatively associated with PRISM scores (correlation coefficient = − 0.260, *P* = 0.012)

There were no significant differences in PRISM scores, temperature, procalcitonin (PCT), lactate levels, white blood cell (WBC) count, ventilation time, length of hospital stay, and length of PICU stay between the septic patients with and without VAD. Differences in the ratio of positive blood culture were not significant. Compared with septic patients without VAD, those patients with VAD had a higher rate of hypoglycemia (4.5% vs. 18.1%; *P* = 0.011) and a lower serum albumin levels (31.64 ± 6.68 vs 28.64 ± 6.25, *P* = 0.004). Hospital mortality and 28-day mortality were not significantly higher in patients with VAD than in those without VAD (*P* > 0.05). Additionally, compared with septic patients without VAD, patients with VAD had a significantly higher incidence of severe sepsis (9.1% vs 24.5%; *P* = 0.013); the incidence of septic shock was also higher in patients with VAD (7.6% vs 21.3%; *P* = 0.019). We also found that patients with VAD had lower platelet (PLT) counts than the patients without VAD (*P* = 0.007) (Table 2). In septic children with VAD, lower vitamin A levels were associated with higher PRISM scores (*r* = − 0.260, *P* = 0.012) (Fig. 2). We also found that VA levels were related with serum albumin levels (*r* = 0.322, *P* < 0.001).

**Table 2:** Clinical characteristics of patients with sepsis stratified by vitamin A status

	Non-VAD *N* = 66	VAD *N* = 94	*P* value
Hypoglycemia, *n* (%)	3 (4.5%)	17 (18.1%)	0.011^b^
Positive blood culture, *n* (%)	6 (9.1%)	8 (8.5%)	0.898^b^
Severe sepsis, *n* (%)	6 (9.1%)	23 (24.5%)	0.013^b^
Septic shock, *n* (%)	5 (7.6%)	20 (21.3%)	0.019^b^
PLT, × 10^9^/L	298.00 (174.00,397.00)	201.50 (104.75,323.25)	0.007^a^
PRISM	10.00 (7.8,16.0)	11.00 (7.0, 17.3)	0.674^a^
Temperature, °C	38.50 (37.60, 39.20)	38.80 (37.80, 39.40)	0.171^a^
PCT, μg/L	3.02 (0.79, 15.12)	3.15 (0.79, 15.69)	0.762^a^
Albumin, g/L	31.64 (6.68)	28.64 (6.25)	0.004^d^
WBC, × 10^9^/L	12.63 (7.01, 16.85)	11.80 (7.89,15.77)	0.438^a^
Lactate, mmol/L	2.70 (1.70, 3.90)	2.45 (1.68, 4.50)	0.967^a^
Ventilation time, hours	24.00 (9.50, 125.00)	22.00 (5.00, 99.50)	0.397^a^
Length of ICU stay, days	8.75 (3.80, 14.00)	5.80 (3.00, 11.75)	0.210^a^
Length of hospital stay, days	16.2 0 (11.30, 20.90)	13.70 (8.45, 23.72)	0.362^a^
Hospital mortality, *n* (%)	2 (3.0%)	5 (5.3%)	0.701^c^
28-day mortality, *n* (%)	3 (4.5%)	8 (8.5%)	0.527^c^

*PLT* platelet, *°C* degree Celsius, *PCT* procalcitonin, *WBC* white blood count

^a^Mann-Whitney *U* test

^b^Chi-squared test

^c^Fisher exact test

^d^Student’s *t* test

The comparisons between patients with and without septic shock are presented in Table 3. The distributions of age, weight, and sex were similar in the two subgroups. Children with septic shock had higher median of PRISM scores, PCT levels, lactate levels, and lower PLT counts than those without shock. WBC counts were lower in the septic shock group, but this difference was not significant. The incidence of hypoglycemia was significantly higher in patients with septic shock than in those without septic shock, while there was no significant difference in the ratio of positive blood culture between the two groups. Children with septic shock suffered longer ventilation times and ICU days and hospital days and have higher mortality both in-hospital and 28-day.

**Table 3:** Comparison between patients with septic shock and without septic shock

	Septic shock *N* = 25	Non-septic shock *N* = 135	*P* value
Age, months	14.00 (1.00, 60.05)	12.00 (3.00, 50.00)	0.841^a^
Weight, kg	8.00 (4.25, 15.50)	7.5 (4.00, 14.50)	0.842^a^
Male, *n* (%)	16 (64.0%)	75 (55.6%)	0.434^b^
PRISM	18.00 (15.0, 34.0)	10.00 (6.00, 14.00)	0.000^a^
Temperature, °C	39.00 (38.20, 39.50)	38.50 (37.70, 39.20)	0.083^a^
PCT, μg/L	42.76 (5.56,100.00)	2.36 (0.70, 9.03)	0.000^a^
PLT, × 10^9^/L	84.00 (48.50, 195.50)	282.0 (157.0, 369.00)	0.000^a^
WBC, × 10^9^/L	10.95 (3.95, 15.79)	12.58 (8.14, 16.50)	0.204^a^
Lactate, mmol/L	5.10 (3.80, 7.90)	2.30 (1.60, 3.40)	0.000^a^
Hypoglycemia, *n* (%)	10 (40%)	10 (7.4%)	0.000^c^
Positive blood culture, *n* (%)	4 (16.0%)	10 (7.4%)	0.237^c^
VAD, *n* (%)	20 (80.0%)	74 (54.8%)	0.019^b^
Ventilation time, hours	182.00 (72.00, 350.00)	20.00 (5.00, 72.00)	0.000^a^
Length of ICU stay, days	12.70 (7.85, 33.95)	5.80 (3.10, 11.50)	0.000^a^
Length of hospital stay, days	18.00 (12.10, 42.35)	14.00 (8.80, 22.00)	0.043^a^
Hospital mortality, *n* (%)	4 (16.0%)	3 (2.2%)	0.012^c^
28-day mortality, *n* (%)	6 (24.0%)	5 (3.7%)	0.002^c^

^a^Mann-Whitney *U* test

^b^Chi-squared test

^c^Fisher exact test

Univariate analysis was performed to compare the patients with septic shock to those without septic shock (Table 4). Variables with *P* < 0.10 were identified as significant contributors to septic shock and included VAD, hypoglycemia, PRISM sores, temperature, PCT, PLT, and lactate levels. Based on the statistically significant differences evident in the univariate analysis, the results of the multivariable regression analysis indicated that VAD (odds ratio (OR) 4.630; 95% confidence interval (CI) 1.027–20.866; *P* = 0.046), PCT (OR 1.029; 95% CI 1.009–1.048; *P* = 0.003), and PRISM scores (OR 1.132; 95% CI 1.009–1.228; *P* = 0.003) were independently associated with septic shock (Table 4). PLT showed a protective effect against septic shock (OR 0.994; 95% CI 0.988–0.999; *P* = 0.045). Univariate and multivariable methods were also used to analyze the risk factors for severe sepsis, and an independent effect of VAD on severe sepsis was identified (Additional file 1 and Additional file 2).

**Table 4:** Regression analysis to identify predictors of septic shock

	Univariate analysis		Multivariable analysis	
	OR (95% CI)	*P* value	OR (95% CI)	*P* value
Age	1.001 (0.993–1.010)	0.804		
Male	1.422 (0.587–3.444)	0.435		
PRISM	1.178 (1.102–1.260)	0.000	1.132 (1.009–1.228)	0.003
Temperature	1.599 (0.991–2.581)	0.054		
PCT	1.037 (1.023–1.052)	0.000	1.029 (1.009–1.048)	0.003
PLT	0.989 (0.984–0.994)	0.000	0.994 (0.988–0.999)	0.045
WBC	0.955 (0.891–1.024)	0.200		
Lactate	1.379 (1.169–1.626)	0.000		
Hypoglycemia	8.333 (2.983–23.277)	0.000		
VAD	3.297 (1.169–9.300)	0.024	4.630 (1.027–20.866)	0.046
Positive blood culture	2.381 (0.683–8.296)	0.173		

## Discussion

In the present study, we found that the majority of patients with sepsis were boys and were younger than 60 months of age. The overall mortality in the study population was 4.4%. Similar findings have been reported by other investigators [10, 19]. Our study demonstrated a significantly higher prevalence of VAD in critically ill children with sepsis, especially in children with severe sepsis and/or septic shock, in the PICU than in approximate-health subjects. VAD is a global health problem. An investigation sponsored by the WHO revealed that nearly 200 million preschool children and 20 million pregnant women were affected by VAD. The investigators also found that 5.2 million preschool children and 9.8 million pregnant women suffered night blindness [18]. In China, according to a nationwide investigation of 8669 children aged 0 to 6 years, the prevalence of VAD was reported as 11.7% [20].

In recent decades, considerable literature has provided evidence that there is an association between VAD and childhood mortality [21–23]. A study with a large population conducted in Indonesia reported that children who did not receive VA capsules in the past 6 months were more likely to be affected by infections [24]. A prospective study with nearly 3000 children indicated that the risk for enteric infections was 2.17-fold higher and the risk for respiratory infections was 2.36-fold higher in children with VAD than in children with an adequate VA status [25]. In addition, our previous findings showed that most of the children with hand, foot, and mouth disease presented VA insufficiency, which was associated with their reduced immunity and more severe illness [8]. Remarkably, most of these studies were performed in Africa and Southeast Asia, suggesting a high prevalence of VAD in these areas, which have been shown in the WHO Global Database on VAD [18].

One of our aims was to reveal the association between VAD and the clinical outcome in children with sepsis. We found that both the hospital mortality and 28-day mortality in septic children with VAD were higher than in those patients without VAD, although the differences were not significant. In addition, our data indicated that lower vitamin A levels were associated with higher PRISM scores and lower albumin levels in septic children. Remarkably, we revealed that patients with VAD were more likely to suffer severe sepsis and septic shock, which can directly lead to poor outcomes. Furthermore, after variable adjustment by the multivariable model, VAD showed an independent association with septic shock and severe sepsis, which involve multiple organs.

Sepsis is characterized by hyperinflammatory in the early period, which may result in sepsis-related multiple organ failure and death. In the present study, VAD was revealed to be independently associated with septic shock and severe sepsis. It is conceivable that VAD may partially contribute to the hyperinflammatory responses in sepsis because of the importance of VA in balancing pro- and anti-inflammatory immunity. VA can enhance anti-inflammatory regulatory T cell differentiation through increasing the expression and phosphorylation of Smad3 and the expression of forkhead box protein 3 (Foxp3), whereas it suppresses the IL-6-driven induction of proinflammatory TH17 cells [4, 26]. It was reported that VA has a dose-dependent antagonistic effect on IL-6, which exerts an important role in the process of systemic inflammatory response syndrome [4, 27]. In addition, in the situation of VAD, the inflammation response was aggravated, which is an unfavorable condition for patients with sepsis in the early phases [28], and downregulated inflammatory responses were found both in human and animal models when they were treated with RA [29]. Furthermore, a negative correlation between VA levels and C-reactive protein levels in a previous study also confirmed that VAD was related to high inflammatory responses [30]. In this study, although there was no significant difference, we found a slightly higher PCT level in patients with VAD than in patients without VAD (Table 2).

Another finding of this study is that PLT counts were negatively associated with septic shock. Similar observations have also been indicated in previous studies [31–33]. In these studies, thrombocytopenia was recognized as a strong negative prognostic marker in patients with sepsis and was associated with illness severity. Interestingly, we found that septic children with VAD had significantly lower PLT levels than children without VAD. There is evidence that all-trans RA can regulate synthetic events in anucleate human platelets through RA receptor α, which is expressed in human platelets [34]. In this aspect, VAD may play another role in sepsis by regulating PLT.

VAD may also play a specific role in the later period of sepsis. In this scenario, the state of immune system dysfunction exposes patients to a high risk of superimposed infections. VAD can impair the barrier function of epithelia covering the digestive, respiratory, and urinary tracts, which is more vulnerable in the condition of sepsis, resulting in an increased risk of infection [1]. In addition, it has been demonstrated that both in humans and animal models, the ability of rebuilding the damaged mucosal integrity could be weakened by VAD [1]. In this situation, the pathogens can penetrate through the mucosal barrier more easily [35, 36]. Furthermore, VAD can lead to the disorder of normal neutrophil development [6] and impair chemotaxis and phagocytosis function, which may diminish the clearance of bacteria in blood [37]. Evidence also indicates that VAD can decrease the capacity of macrophages in bacteria-killing and phagocytosing actions [38]. Those findings may be explanations for why children with VAD had a lower WBC count than those without VAD in the current study.

It seems that there are extensive interactions between VAD and sepsis based on the current study. Further studies are needed to help deeply understand the relation between VAD and sepsis. In addition, interventional studies of vitamin A supplementation (VAS) are also worthwhile in fighting with sepsis since VA may be helpful in alleviating the uncontrolled inflammatory responses during the hyperinflammatory phases and restoring immune function during the immunosuppressive phases. In addition, VAS is suggested by the WHO as a beneficial, cost-effective intervention to prevent morbidity and mortality in children. It has been reported that VAS could reduce mortality by as much as 34% in children with VAD [39]. According to the Cochrane database, VAS could decrease the risk of all-cause mortality by 24% [40].

Infectious diseases could result in impaired nutrient absorption and utilization and direct nutrient losses [41–43] along with common conditions of inadequate nutritional supplementation in the ICU. In this regard, urinary retinol loss of VA can be substantial. Higher disease severity is associated with higher concentrations of urinary retinol [44, 45]. In addition, although no data were available on VA status in the ICU, a prospective cohort study revealed that vitamin D status on day 7 in the ICU was significantly lower than on admission [46]. Therefore, when the disease continues to persist, the prevalence of VAD in children with sepsis may be higher than that indicated in the current study.

Our study has several limitations. First, VA status before admission was not available in all subjects. It is not certain whether the result represents preexisting VAD since infection could decrease VA levels [1]. Second, VA concentrations were evaluated only one time shortly after admission. Therefore, the variations in VA status during the PICU period were largely unknown. In this regard, a range of fluctuation in the serum VA status may be a determinant factor in the interaction between VAD and sepsis to some degree. Third, the current study had a small simple size, and all patients were recruited from one medical center, both of which may result in selection bias.

## Conclusion

In conclusion, we found that VAD was associated with severe sepsis, septic shock, and higher PRISM score. Our study indicated that VAD may be a marker of mortality in critically ill children with sepsis. These findings suggest that investigators should pay more attention to VAD in children with sepsis who are admitted into the PICU, especially children with severe sepsis and/or septic shock. VAS may be a potential therapy for sepsis according to its important role in immune modulation. Currently, there are no data available on this issue. Further studies are needed to evaluate and verify this possibility, with the aim of gaining a greater understanding of the effects of VA intake on the outcomes in children with sepsis.

## Additional files

Additional file 1: **Table S1.** Comparison between patients with severe sepsis and without severe sepsis. (DOCX 13 kb)

Additional file 2: **Table S2.** Regression analysis to identify predictors of severe sepsis. (DOCX 12 kb)

## Data Availability

The datasets used for the analysis in the current study are available from the corresponding author on reasonable request.

## Abbreviations

CI: Confidence interval
EV71: Enterovirus 71
IFN-α: Interferon-α
IgM: Immunoglobulin M
IL-6: Interleukin 6
LBW: Low birth weight
NK cell: Natural killer cell
OR: Odds ratio
PCT: Procalcitonin
PICU: Pediatric intensive care unit
PLT: Platelet
PRISM: Pediatric Risk of Mortality
RA: Retinoic acid
Th17: T helper 17 cell
Treg: Regulatory T cell
VA: Vitamin A
VAD: Vitamin A deficiency
WBC: White blood cell count
WHO: World Health Organization
