# Supplementary material for: Vitamin A deficiency in critically ill children with sepsis
Source: Crit Care. 2019 Aug 1;23:267. doi: 10.1186/s13054-019-2548-9 (PMC6670191; doi:10.1186/s13054-019-2548-9)
Supplement: Supplementary file 1 — Table S1. Comparison between patients with severe sepsis and without severe sepsis. (DOCX 13 kb) [file 13054_2019_2548_MOESM1_ESM.docx]

Additional file 1: Table S1 Comparison between Patients with Severe Sepsis and without Severe Sepsis

|  | Severe sepsis  N=29 | Non-severe sepsis  N=131 | *P* value |
| --- | --- | --- | --- |
| Age, months | 12.00 (1.00, 48.00) | 12.00 (3.00, 56.00) | 0.842 ^a^ |
| Weight, kg | 8.00 (3.33, 14.25) | 7.50 (4.00, 15.00) | 0.553 ^a^ |
| Male, n (%) | 19 (65.5%) | 72 (55.0%) | 0.299 ^b^ |
| PRISM | 18.00 (15.00, 33.50) | 10.00 (6.00, 13.00) | 0.000 ^a^ |
| Temperature, ℃ | 39.00 (37.80, 39.45) | 38.50 (37.70, 39.20) | 0.212 ^a^ |
| PCT, μg/L | 13.62 (3.13, 100.00) | 2.61 (0.70, 10.05) | 0.000 ^a^ |
| PLT, ×10^9^/L | 90.00 (58.50, 195.50) | 283.00 (158.00, 369.00) | 0.000 ^a^ |
| WBC, ×10^9^/L | 10.95 (4.85, 15.45) | 12.58 (8.14, 16.60) | 0.211 ^a^ |
| Lactate, mmol/L | 5.10 (3.40, 7.90) | 2.20 (1.60, 3.40) | 0.000 ^a^ |
| Hypoglycemia, n (%) | 12 (41.4%) | 8 (6.1%) | 0.000 ^c^ |
| VAD, n (%) | 23 (79.3%) | 71 (54.2%) | 0.013 ^b^ |
| Positive blood culture, n (%) | 4 (13.8%) | 10 (7.6%) | 0.285 ^c^ |
| Ventilation time, hours | 185.00 (77.00, 351.50) | 20.00 (4.00, 72.00) | 0.000 ^a^ |
| Length of ICU stay, days | 12.70 (7.85, 30.10) | 5.50 (3.00, 10.90) | 0.000 ^a^ |
| Length of hospital stay, days | 18.00 (11.20, 38.15) | 14.00 (8.80, 21.00) | 0.049 ^a^ |
| Hospital mortality, n (%) | 5 (17.2%) | 2 (1.5%) | 0.002 ^c^ |
| 28-day mortality, n (%) | 7 (24.1%) | 4 (3.1%) | 0.001 ^c^ |

^a^ Mann-Whitney U test.

^b^ Chi-squared test.

^c^ Fisher exact test.
