# Supplementary material for: Vitamin A deficiency in critically ill children with sepsis
Source: Crit Care. 2019 Aug 1;23:267. doi: 10.1186/s13054-019-2548-9 (PMC6670191; doi:10.1186/s13054-019-2548-9)
Supplement: Supplementary file 2 — Table S2. Regression analysis to identify predictors of severe sepsis. (DOCX 12 kb) [file 13054_2019_2548_MOESM2_ESM.docx]

Additional file 2: Table S2. Regression Analysis to Identify Predictors of Severe Sepsis

|  | Univariate analysis | | Multivariable analysis | |
| --- | --- | --- | --- | --- |
|  | OR (95% CI) | *P* value | OR (95% CI) | *P* value |
| Age | 0.999 (0.991, 1.007) | 0.831 |  |  |
| Male | 1.557 (0.672, 3.605) | 0.301 |  |  |
| PRISM | 1.204 (1.119, 1.295) | 0.000 | 1.198 (1.102-1.301) | 0.000 |
| Temperature | 1.276 (0.824, 1.977) | 0.275 |  |  |
| PCT | 1.032 (1.019, 1.046) | 0.000 | 1.027 (1.009-1.045) | 0.003 |
| PLT | 0.991 (0.987, 0.995) | 0.000 |  |  |
| WBC | 0.957 (0.896, 1.021) | 0.1.85 |  |  |
| Lactate | 1.502 (1.242, 1.817) | 0.000 |  |  |
| Hypoglycemia | 10.853 (3.881, 30.350) | 0.000 |  |  |
| VAD | 3.239 (1.238, 8.477) | 0.017 | 5.193 (1.391-19.390) | 0.014 |
| Positive blood culture | 1.936 (0.562,6.669) | 0.295 |  |  |
